# The Altered Renal and Hepatic Expression of Solute Carrier Transporters (SLCs) in Type 1 Diabetic Mice

**DOI:** 10.1371/journal.pone.0120760

**Published:** 2015-03-19

**Authors:** Chenghao Xu, Ling Zhu, Ting Chan, Xiaoxi Lu, Weiyong Shen, Mark C. Gillies, Fanfan Zhou

**Affiliations:** 1 Faculty of Pharmacy, The University of Sydney, NSW 2006, Sydney, Australia; 2 Retinal Therapeutics Research Group, Save Sight Institute, The University of Sydney, Sydney, NSW 2000, Australia; Emory University, UNITED STATES

## Abstract

Diabetes mellitus is a chronic metabolic disorder that significantly affects human health and well-being. The Solute carrier transporters (SLCs), particularly the Organic anion/cation transporters (Oats/Octs/Octns), Organic anion transporting polypeptides (Oatps) and Oligopeptide transporters (Pepts) are essential membrane proteins responsible for cellular uptake of many endogenous and exogenous substances such as clinically important drugs. They are widely expressed in mammalian key organs especially the kidney and liver, in which they facilitate the influx of various drug molecules, thereby determining their distribution and elimination in body. The altered expression of SLCs in diabetes mellitus could have a profound and clinically significant influence on drug therapies. In this study, we extensively investigated the renal and hepatic expression of twenty essential SLCs in the type 1 diabetic Ins2^Akita^ murine model that develops both hyperglycemia and diabetes-related complications using real-time PCR and immunoblotting analysis. We found that the renal expression of mOatp1a1, mOatp1a6, mOat1, mOat3, mOat5, mOct2 and mPept2 was decreased; while that of mPept1 was increased at the mRNA level in the diabetic mice compared with non-diabetic controls. We found up-regulated mRNA expression of mOatp1a4, mOatp1c1, mOctn2, mOct3 and mPept1 as well as down-regulation of mOatp1a1 in the livers of diabetic mice. We confirmed the altered protein expression of several SLCs in diabetic mice, especially the decreased renal and hepatic expression of mOatp1a1. We also found down-regulated protein expression of mOat3 and mOctn1 in the kidneys as well as increased protein expression of mOatp1a4 and mOct3 in the livers of diabetic mice. Our findings contribute to better understanding the modulation of SLC transporters in type 1 diabetes mellitus, which is likely to affect the pharmacokinetic performance of drugs that are transported by these transporters and therefore, forms the basis of future therapeutic optimization of regimens in patients with type 1 diabetes mellitus.

## Introduction

Diabetes mellitus is a chronic metabolic disorder, which not only impacts on human health and well-being, but also results in significant social and economic consequences. As to the fact sheets of the World Health Organization, diabetes mellitus is among the top 10 causes of death and there are 347 million people suffering from this disease world-widely (data collected in Nov. 2014). This disease is caused by under-production or ineffective usage of insulin in body, which then lead to the deregulation of blood glucose. Hyperglycemia (high blood glucose level) over a period of time can damage the blood vessels and nerves as well as many other body systems, which consequently cause life threatening complications such as impairment of immune system, retinopathy, nephropathy and cardiovascular diseases [1].

Due to different pathogenesis, diabetes mellitus can be classified into several subtypes with Type 1 diabetes mellitus (T1DM) and Type 2 diabetes mellitus (T2DM) representing more than 90% of cases. T1DM is non-preventable and insulin dependent, which is often diagnosed in childhood. Patients with T1DM require invasive daily administration of insulin. T2DM is insulin independent and primarily adult-onset. Although T2DM influences a larger population than T1DM, it is preventable with healthy life styles. Both subtypes have common symptoms; however, those of T1DM are often more severe [2, 3]. In all types of diabetes mellitus, gene expression changes have been widely observed, which not only contribute to disease progression but also impact on clinical outcomes of pharmaceutical therapies [4–6].

Solute carrier transporters (SLCs) are a superfamily of membrane proteins responsible for cellular uptake of a diverse range of substances including hormones, steroids, toxins and many clinically important drugs [7]. Among all the SLC members, the Organic anion transporting polypeptides (Oatps), Organic anion/cation transporters (Oats/Octs) and Oligopeptide transporters (PepTs) represent the most important SLC subfamilies involved in drug performance [7–9]. These transporters are widely expressed in mammalian key organs especially the liver and kidney [10, 11], in which tissues they are responsible for uptake of drug molecules into cells and therefore, greatly impact on drug distribution and elimination [12]. The function and expression of SLC transporters in specific tissues profoundly influence therapeutic outcomes and toxicities.

Literature has reported the altered expression of SLC transporters under disease status including diabetes mellitus and obesity [13, 14]. In the study of Grover et al., renal expression of rOct1 and rOct2 was found to be decreased together with the disease progression in the streptozotocin-induced diabetic rats [15]. In the later study investigating the renal and hepatic expression of rOats/rOcts in a diet- and streptozotocin-injected T2DM model, Nowicki et al. revealed the decreased expression of rOct2 in the kidneys of diabetic animals together with the elevated expression of rOat2 [14]. Recently, the renal expression of rOat3 was found to be subject to the molecular regulation of insulin in the streptozotocin-induced diabetic rats [16, 17]. The report of Cheng et al. demonstrated the down-regulated renal expression of mOatp1a1 and mOat2 in a murine model of obesity and T2DM [18]. Observation made by More et al. also showed the impaired expression of mOatp1a1 and mOatp1b2 in the livers and kidneys of the severe T2DM mice [19]. In addition, elevated abundance of rPept1 was identified in the intestine of streptozotocin-induced rats, which finding may have nutritional and pharmacological implications due to the role of Pept1 played in transporting dietary di- or tri-peptides as well as many clinical drugs [20, 21]. However, no expressional changes of rPept1 and rPept2 were identified in the kidneys of streptozotocin-induced diabetic rats [22]. Upon from the studies mentioned above, the expressional profile of SLC transporters in diseases, particularly in T1DM remains largely unknown.

In this study, the renal and hepatic expression of twenty essential SLC transporters was investigated in the non-obese, insulin-deficient Ins2Akita murine model at both mRNA and protein levels. As mentioned above, streptozotocin-injected animals have been widely used previously; however, studies in these invasively induced diabetic animals provided evidence for differential susceptibility to the development of kidney injury among genetically distinct mouse lines [23–25]. In addition, streptozotocin has been found to be toxic to various other tissues, which may complicate the interpretation of results [25]. In contrast, Ins2Akita mouse with a dominant point mutation in the Insulin 2 gene on chromosome 7 leading to pancreatic β-cell apoptosis and hyperglycemia appears to be a more preferable diabetic murine model, specifically in representing T1DM. Ins2Akita mouse develops diabetes at approximately 4 weeks after birth with almost 100% penetrance. This murine model possesses proper renal phenotype and favorable hyperglycemia characteristics [25–28]. Moreover, complications commonly related with diabetes in human such as hypertension, heart failure and cardiac hypertrophy have been found in this murine model [25]. Overall, Ins2Akita mouse model gives more advantages over chemical-induced rodent models, which is a more similar-to-human experimental platform in diabetic studies.

## Materials and Methods

### Materials

The resource for specific antibodies against each SLC transporter is listed in Table 1. The horseradish peroxidase-conjugated donkey anti-goat IgG was obtained from Sapphire Biosciences (Waterloo, NSW, Australia). The horseradish peroxidase-conjugated goat anti-rabbit IgG was purchased from Sigma-Aldrich (Castle Hill, NSW, Australia). Unless otherwise stated, all other chemicals and biochemicals were purchased from Sigma-Aldrich (Castle Hill, NSW, Australia).

**Table 1:** Primary antibody information.

Transporters	Primary Antibody Resource			Primary Antibody Dilution
	Company	Catalogue Number	Specification	
mOat1*	Bioss	bs-0607R	Rabbit/Polyclonal	1:1000
mOat2**	Abcam	ab191018	Rabbit/Polyclonal	1:1000
mOat3*	Bioss	bs-0609R	Rabbit/Polyclonal	1:1000
mOat5	Santa Cruz	Sc-109029	Goat/ Polyclonal	1:200
mOctn1	Santa Cruz	Sc- 19819	Goat/ Polyclonal	1:1000
mOctn2*	Alpha Diagnostic	Octn21-A	Rabbit/Polyclonal	1:1000
mOct2*	Bioss	bs-1077R	Rabbit/Polyclonal	1:1000
mOct3**	Abcam	ab191446	Rabbit/Polyclonal	1:1000
mOatp1a1	Santa Cruz	Sc- 47265	Goat/ Polyclonal	1:1000
mOatp1a4*	Santa Cruz	Sc- 18436	Goat/ Polyclonal	1:1000
mOatp1c1**	Abcam	ab 83972	Rabbit/Polyclonal	1:1000
mPept1	Santa Cruz	Sc-20653	Rabbit/Polyclonal	1:200
mPept2	Santa Cruz	Sc- 19920	Goat/ Polyclonal	1:200

Secondary: Horseradish peroxidase-conjugated goat anti-rabbit IgG (1:10,000; Sigma-Aldrich, Castle Hill, NSW, Australia, Cat. No: A0545) and Donkey anti-goat IgG-HRP (1:10,000; Sapphire Biosciences, Waterloo, NSW, Australia, Cat. No: Sc-2020)

*: Antibodies have been used in the previous studies [29, 30].

**: Antibodies have been validated by manufacturers.

### Animals

Our animal studies were conducted in accordance with the New South Wales Animals Act (1985) with the approval from The University of Sydney Animal Ethics Committee (Permit number: K17/1–2013/3/5884). All surgery was performed under sodium pentobarbital anesthesia and all efforts were made to minimize suffering.

As mentioned previously [31], the Ins2Akita mice were obtained from The Jackson Laboratory (Bar Harbor, ME, USA). Because female mice develop diabetes more slowly and less stably, male mice were preferably used in the current study [27]. The male Ins2Akita mice heterozygous for the Ins2Akita allele are the diabetic group (n = 7); while those homozygous for the wild type Ins2 allele were used as control (n = 7). Genotyping was conducted to determine the Ins2Akita allele or the wild type Ins2 gene [28, 31]. Mice with blood glucose level consistently≥13.8 mmol/l were considered as fully developed diabetes [32]. Changes of body weight and blood glucose levels (using Accu-Chek Performa, Roche, Germany) were monitored all the time. No supplemental insulin was given to all the mice. After euthanizing mice (age = 12 weeks) with CO2, the kidneys and livers were removed, snap-frozen in liquid nitrogen and stored in -80°C freezer for further studies.

### RNA Extraction and Quantification

Total RNA from the livers and kidneys was isolated by phenol-chloroform extraction using Trizol Reagent (Invitrogen, Mount Waverley, Victoria, Australia) according to the manufacturer’s protocol. The RNA was quantified by measuring its absorbance at 280nm in a UV-visible spectrophotometer (NanoDrop ND 1000; Thermo Fisher Scientific, Scoresby, VIC, Australia). Agarose gel electrophoresis was also used to check RNA integrity.

### Real time reverse transcription polymerase chain reaction (RT-PCR)

First-strand cDNA was synthesized using the high capacity cDNA reverse transcription kit (Life Technologies, Mulgrave, VIC, Australia). Expression of mRNAs corresponding to each SLC transporter gene was assessed by SYBR green quantitative PCR with the ABI 7500 sequence detection system (Invitrogen, Mount Waverley, VIC, Australia). The gene-specific primers targeting the SLC genes and β-actin are listed in the Table 2. After each PCR, a melting curve analysis was performed to confirm product specificity. Transporter mRNA levels were normalized to β-actin and the relative expression was determined using the 2-ΔΔCT method comparing to that of mOatp1a1 [31, 33, 34]. Data analysis was performed using the Relative Expression Software Tool.

**Table 2:** SLC gene specific real-time PCR primers used in this study.

Transporter	Forward Primer 5′ to 3′	Reverse Primer 5′ to 3′
mOat1	CTGATGGCTTCCCACAACAC	GTCCTTGCTTGTCCAGGGG
mOat2	CAACTGCGGAATCTGGTGCT	ATCAGGCAGGGCACAATGATG
mOat3	ATGACCTTCTCCGAGATTCTGG	GTGGTTGGCTATTCCGAGGAT
mOat5	AAATGCAGATCCTGCGTGTATT	CCTAAAGCAGTTGCCCTGATTA
mOctn1	TGGTATGTCAGTCGTGTTCCT	AGCCCCATCGCAGAGAAGT
mOctn2	ACTGTGCCAGGGGTGCTAT	GCAACTGAGGCTTCGTAGAAT
mOctn3	CGTGGGTGTGCTCTTAGGC	TTGTATGAAGCTGAATCCGGTG
mOct1	GACGCCTGGAAAGTGGACC	GCAACATGGATGTATAGTCTGG
mOCt2	CCAGTGCATGAGGTATGAGGT	CTGAAACAGGTCCAGCATCCA
mOct3	CAGCCCGACTACTATTGGTGT	TGAGCTGGTATTAGTGGCTTCC
mOatp1a1	GTGCATACCTAGCCAAATCACT	CCAGGCCCATAACCACACATC
mOatp1b2	GGGAACATGCTTCGTGGGATA	GGAGTTATGCGGACACTTCTC
mOatp1a4	GCTTTTCCAAGATCAAGGCATTT	CGTGGGGATACCGAATTGTCT
mOatp1a5	CATGCTTCTCATCCTGACAAGT	GAGGACGACCTCTGAAGTGG
mOatp1a6	ACAGGGTCAGGTGCTTTGC	ATCACCAAAAGGTTACCCATCTC
mOatp2b1	CTCAGGACTCACATCAGGATGC	CTCTTGAGGTAGCCAGAGATCA
mOatp4a1	GCGATGGGGGACACACATTT	CTGTCTGGCTACTCCGCTTC
mOatp1c1	GGGCCATCCTTTACAGTCGG	CCTTCTCTCTATCTGAGTCACGG
mPept1	CCGGCACACCCTTCTAGTG	TGGCGTTGTGACTGGTGAC
mPept2	AAAGCGACAACATTGGCTAGA	AAATCCCAAATCGCCATCCAT
β-actin	TTCTTTGCAGCTCCTTCGTT	ATGGAGGGGAATACAGCCC

### Electrophoresis and immunoblotting

Tissue sample was homogenized and lysed with lysis buffer (10 mM Tris, 150 mM NaCl, 1 mM EDTA, 0.1% sodium dodecyl sulfate, 1% Triton X-100, that contained the protease inhibitors phenylmethylsulfonyl fluoride, 200 mg/mL, and leupeptin, 3 mg/mL, pH 7.4). After centrifugation at 4°C for 10 minutes, supernatants were transferred into tubes. Bradford assay was used to measure protein concentration. Equivalent quantities of protein lysates from each sample were denatured at 55°C for 30 min in Lammli reducing buffer as described before [35–40]. Protein samples were loaded onto 7.5% polyacrylamide mini gels and electrophoresed (Bio-Rad, Gladesville, New South Wales, Australia). Proteins were transferred to polyvinylidene fluoride membrane in an electroelution cell (Bio-Rad, Gladesville, New South Wales, Australia). The membrane was blocked for 1 hour with 5% bovine serum albumin in PBS-Tween (137 mM NaCl, 2.7 mM KCl, 4.3 mM Na2HPO4, 1.4 mM KH2PO4 and 0.05% Tween 20, pH 7.4). The blot was then washed with PBS-Tween thoroughly and incubated overnight with primary antibody at 4°C. On the next day, the membrane was washed with PBS-Tween, incubated with secondary antibody for one hour at room temperature. The membrane was washed with PBS-Tween and then incubated with the Immobilon Western Chemiluminescent HRP Substrate (Merck; Kilsyth, VIC, Australia). In all experiments, membranes were re-probed for β-actin (1:1,000; Cat. No: 4967; Genesearch, Arundel, Qld, Australia). The dilutions of primary and secondary antibodies used in this study are listed in the Table 1.

### Statistics

Data are presented throughout as mean ± S.E. The unpaired t-test was used to evaluate differences between two sets of normally distributed data.

## Results

### Altered mRNA expression of SLC transporters in the kidneys and livers of diabetic mice

We assessed the mRNA expression of twenty SLC genes including mOat1, mOat2, mOat3, mOat5, mOatp1a1, mOatp1a4, mOatp1a5, mOatp1a6, mOatp4a1, mOatp1b2, mOatp2b1, mOatp1c1, mOct1, mOct2, mOct3, mOctn1, mOctn2, mOctn3, mPept1 and mPept2 in the kidneys and livers of diabetic and control mice, which range widely cover the most studied Oatps, Oats/Octs/Octns and Pepts so far. Our initial RT-PCR analysis (data processed through the web-based RefFinder software: http://www.leonxie.com/referencegene.php?type=reference) showed that β-actin is the most conserved gene in both groups compared to the other five commonly used housekeeping genes including Gapdh, 18sRNA, β2-microglobulin, glucuronidase β, heat-shock protein 90α (data not shown); therefore, the relative gene expression of SLC transporters in the kidneys and livers was then normalized to that of β-actin (Table 3 and 4). The basal expression of SLCs in the control mice was indicated as relative to that of mOatp1a1, because mOatp1a1 has relatively high expression in both the kidney and liver. The changes of SLC expression in the diabetic group were expressed as their folds to those in control. Unpaired t-test was applied to calculate the statistical significance of gene expressions between these two groups.

**Table 3:** The mRNA expression of SLC transporters in the kidneys of diabetic and control mice.

Transporter	Relatively gene expression in the control mice	SLC gene expression in the diabetic mice (fold of control)		P-value
		Expression	Standard Error	
mOatp1a1	1.00	0.34	0.21–0.57	0.001***
mOatp1a4	1.95x10^-3^	0.75	0.51–1.14	0.100
mOatp1a5	3.05x10^-5^	0.75	0.06–5.96	0.735
mOapt1a6	1.25x10^-1^	0.56	0.43–0.72	0.000***
mOatp4a1	3.91x10^-3^	1.10	0.66–1.98	0.773
mOatp1b2	4.88x10^-4^	0.76	0.24–2.54	0.612
mOatp2b1	3.13x10^-2^	0.97	0.78–1.24	0.786
mOatp1c1	1.57x10^-2^	0.99	0.59–1.41	0.984
mOat1	2.00	0.70	0.58–0.81	0.000***
mOat2	0.50	0.34	0.21–0.51	0.004**
mOat3	1.00	0.53	0.37–0.74	0.001***
mOat5	6.25x10^-2^	0.58	0.44–0.80	0.001***
mOctn1	7.81x10^-3^	0.64	0.38–1.02	0.048*
mOctn2	0.50	0.84	0.65–1.06	0.090
mOctn3	9.17x10^-4^	0.94	0.77–1.15	0.427
mOct1	0.50	0.76	0.50–1.06	0.093
mOct2	0.50	0.60	0.49–0.76	0.000***
mOct3	1.22x10^-4^	0.99	0.28–3.10	0.987
mPept1	1.95x10^-3^	1.54	1.15–1.94	0.002*
mPept2	6.25x10^-2^	0.58	0.43–0.79	0.001***

*: P<0.05

**, P<0.01

***, P<0.001

**Table 4:** The mRNA expression of SLC transporters in the livers of diabetic and control mice.

Transporter	Relatively gene expression in the control mice	SLC gene expression in the diabetic mice (fold of control)		P-value
		Expression	Standard Error	
mOatp1a1	1.00	0.15	0.09–0.58	0.000***
mOatp1a4	3.13x10^-2^	2.51	1.14–5.30	0.007**
mOatp1a5	7.63x10^-6^	0.50	0.20–1.32	0.072
mOapt1a6	-	-	-	-
mOatp4a1	6.10x10^-5^	0.83	0.34–1.85	0.627
mOatp1b2	2.00	0.62	0.25–1.24	0.158
mOatp2b1	0.25	0.97	0.73–1.25	0.753
mOatp1c1	1.22x10^-4^	1.97	1.12–3.21	0.019*
mOat1	-	-	-	-
mOat2	6.25x10^-2^	0.93	0.47–1.73	0.794
mOat3	1.53x10^-5^	0.47	0.12–1.90	0.221
mOat5	-	-	-	-
mOctn1	2.44x10^-4^	1.18	0.76–1.85	0.394
mOctn2	3.13x10^-2^	4.30	2.38–7.57	0.000***
mOctn3	6.10x10^-5^	1.02	0.75–1.35	0.886
mOct1	-	-	-	-
mOct2	-	-	-	-
mOct3	9.77x10^-4^	5.09	3.01–11.19	0.000***
mPept1	1.53x10^-5^	6.19	3.47–12.17	0.003**
mPept2	1.95x10^-3^	0.56	0.18–1.60	0.202

-: Undetermined

*: P<0.05

**, P<0.01

***, P<0.001

Among the eight mOatps assessed in this study, the expression of mOatp1a1 and mOatp1a6 in the diabetic group was decreased to ~0.34 and ~0.56 fold of control, respectively (Table 3). All four members of mOats demonstrated significantly down-regulated expression in diabetic mice (ranged from ~0.34 to ~0.70 fold of control). In addition, mOct2 mRNA expression was decreased to ~0.60 fold of control and that of mOctn1 was moderately decreased in diabetic mice. Noteworthy, mPept1 expression in the kidneys of diabetic mice was increased to ~1.54 folds of control; while that of mPept2 was significantly decreased to ~0.58 fold of control.

Pronounced reduction of mOatp1a1 expression was observed in the livers of diabetic mice; however, that of mOatp1a4 and mOatp1c1 in the diabetic group was increased to ~2.51 and ~1.97 folds of control, respectively (Table 4). In addition, the expression of mOctn2, mOct3 and mPept1 was markedly up-regulated in the livers of diabetic mice (Table 4).

### Modulated protein expression of SLC transporters in the kidneys and livers of diabetic mice

An altered gene expression is not necessarily associated with a modulated protein expression. We further assessed the protein expression of the SLC transporters with altered mRNA levels in the kidneys and livers of diabetic mice and compared to that of control. The relative expression of transporters (normalized against β-actin) in both groups was analyzed with unpaired t-test program of Graphpad Prism 5.0 software. Due to the unavailability of antibodies against mOatp1a6 and mOatp2b1, we were unable to include these two transporters in this part of the study.

**Fig 1:**
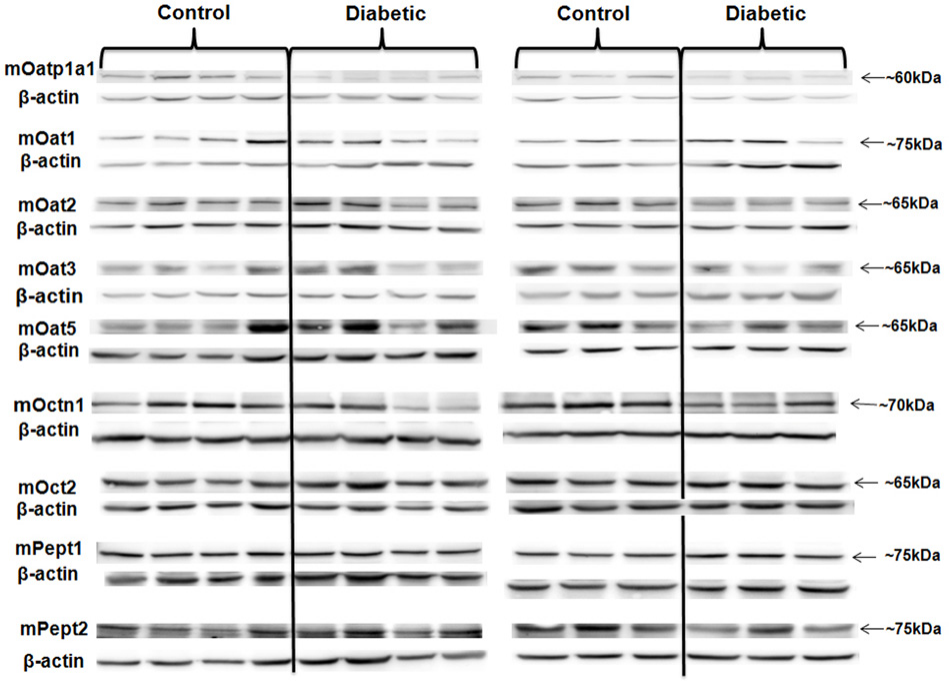
The protein expression of the SLC transporters with altered mRNA expression in the kidneys of diabetic mice compared to that of control. As described in Methods, kidney tissue samples of both control and diabetic mice were lysed. Protein samples were denatured and preceded to electrophoresis. The immunoblots were then probed with specific antibodies of SLC transporters. The same blots were also probed with anti-β-actin antibody. The expression of β-actin was used as normalization control in all the experiments. Each experiment was repeated three times with the representative blot shown in the figure. Diabetic group (n = 7 mice); control group (n = 7 mice).

**Fig 2:**
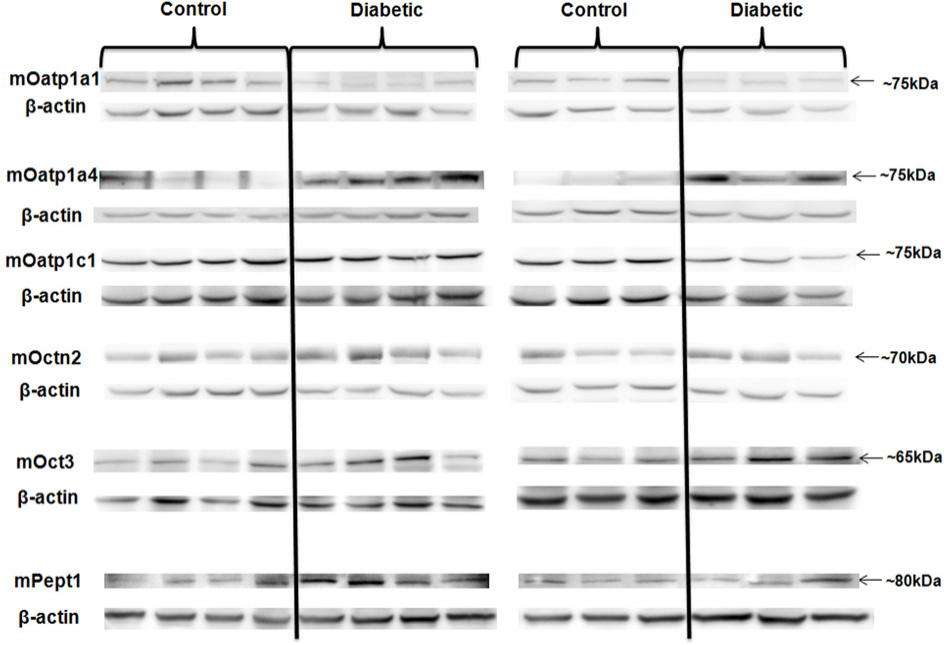
The protein expression of the SLC transporters with altered mRNA expression in the livers of diabetic mice compared to that of control. As described in Methods, liver tissue samples of both control and diabetic mice were lysed. Protein samples were denatured and preceded to electrophoresis. The immunoblots were then probed with specific antibodies of SLC transporters. The same blots were also probed with anti-β-actin antibody. The expression of β-actin was used as normalization control in all the experiments. Each experiment was repeated three times with the representative blot shown in the figure. Diabetic group (n = 7 mice); control group (n = 7 mice).

In the kidneys of diabetic mice, the protein expression of mOatp1a1, mOat3 and mOctn1 was significantly reduced compared with that of control; while the protein expression of all the other six SLC transporters assessed remained unchanged (Fig. 1 and Table 5). Similarly, the protein expression of mOatp1a1 in the livers of diabetic mice was decreased pronouncedly; in contrast, that of mOatp1a4 and mOct3 was up-regulated significantly (Fig. 2 and Table 6). The changes of mOatp1c1, mOctn2 and mPept1 levels were not statistically significant.

**Table 5:** The densitometry analysis of the protein expression of SLC transporters in the kidneys of diabetic and control mice.

Transporter	Relatively protein expression in the control mice	Relatively protein expression in the diabetic mice	Expressional change	P-value
mOatp1a1	1.86 ± 0.22	0.90 ± 0.13	down regulation	0.003 **
mOat1	1.18 ± 0.09	0.24 ± 0.15	unchanged	0.149
mOat2	0.40 ± 0.11	0.50 ± 0.07	unchanged	0.452
mOat3	1.32 ± 0.27	0.47 ± 0.14	down regulation	0.016 *
mOat5	0.80 ± 0.17	0.69 ± 0.16	unchanged	0.628
mOctn1	0.74 ± 0.07	0.33 ± 0.11	down regulation	0.010 *
mOct2	0.73 ± 0.06	0.64 ± 0.08	unchanged	0.420
mPept1	1.31 ± 0.17	1.41 ± 0.23	unchanged	0.724
mPept2	0.29 ± 0.05	0.29 ± 0.08	unchanged	0.976

The relative density of the bands of each SLC transporter was normalized to that of β-actin and analysed with unpaired *t*-test program of Graphpad Prism 5.0 software. The pooled data of all the experimental repeats was included in the analysis. Diabetic group (n = 7 mice); control group (n = 7 mice).

*: P<0.05

**, P<0.01

***, P<0.001

**Table 6:** The densitometry analysis of the protein expression of SLC transporters in the livers of diabetic and control mice.

Transporter	Relatively protein expression in the control mice	Relatively protein expression in the diabetic mice	Expressional change	P-value
mOatp1a1	0.80 ± 0.07	0.44 ± 0.05	down regulation	0.001 **
mOatp1a4	0.98 ± 0.29	2.71 ± 0.23	up regulation	0.001 **
mOatp1c1	0.94 ± 0.07	0.99 ± 0.13	unchanged	0.741
mOctn2	2.52 ± 0.16	2.72 ± 0.13	unchanged	0.361
mOct3	0.22 ± 0.03	0.39 ± 0.06	up regulation	0.031 *
mPept1	0.44 ± 0.03	0.52 ± 0.03	unchanged	0.068

The relative density of the bands of each SLC transporter was normalized to that of β-actin and analysed with unpaired *t*-test program of Graphpad Prism 5.0 software. The pooled data of all the experimental repeats was included in the analysis. Diabetic group (n = 7 mice); control group (n = 7 mice).

*: P<0.05

**, P<0.01

***, P<0.001

## Discussion

It is well known that diabetes mellitus is a major health concern world widely, which results in a range of short- and long-term health complications that are the major causes of associated morbidity and mortality in people [1]. Liver and kidney diseases often occur as consequent complications of diabetes mellitus. Responses to drug therapies often differ between diabetic and non-diabetic populations due to impaired kidney and liver functions under disease status [41–46]. It is plausible that such therapeutic variation could be a consequence of altered function and expression of drug metabolism enzymes as well as membrane transporters in the kidney and liver [47, 48].

Solute carrier transporters, particularly Oatps, Oats/Octs/Octns and Pepts are important membrane influx transporters responsible for the cellular uptake of many endogenous substances as well as clinically important drugs. Their functions profoundly impact on the absorption, distribution and elimination of molecules especially pharmaceutical agents [49]. Previously, altered expression of SLC transporters have been reported in diseases such as obesity and diabetes mellitus [13–22]. However, due to the limitations of chemical-induced diabetic animal models used in these studies, the interpretation of results might be compromised. As described in the introduction, the genetically modified Ins2Akita mouse model possesses proper renal phenotype and favorable hyperglycemia characteristics together with many commonly occurred diabetic complications; therefore, it is more preferred to be used in diabetic studies, particularly in representing the pathological conditions of T1DM. And a number of studies have already been conducted using the Ins2Akita mouse model to investigate the disease management of T1DM [50–52].

Our study extensively evaluated the gene and protein expression of twenty essential SLC transporters in the control and diabetic Ins2Akita mice. Our data revealed significantly down-regulated expression of mOatp1a1 in both the livers and kidneys as well as the up-regulated expression of mOatp1a4 in the livers of diabetic mice (Table 3–6, Fig. 1–2), which observation agrees with the previous report [19]. The suggested deficit of mOatp1a1 and mOatp1a4 in diabetic mice may have profound influence on pharmaceutical treatment in diabetic patients, since these transporters have been shown to greatly influence the pharmacokinetic performance of many clinically important drugs such as doxorubicin [53], rosuvastatin [54, 55], paclitaxel [56], methotrexate [56] and bosentan [57]. It is plausible that the impaired expression and function of Oatps in diabetes might lead to unsatisfied efficacy and/or unexpected toxicity of these drugs. In addition, the research trend in screening the potential anti-diabetic candidate molecules often favors the recognitions of Oatps [58, 59]. Thus, the altered expression and function of these transporters should be considerably included in the drug screening strategy targeting at diabetes. Furthermore, an increased susceptibility to cholestatic liver injury has also been demonstrated in mice with mOatp1a1 dysfunction [60]; therefore, the deregulated mOatp1a1 observed in our study might potentially contribute to elucidate the pathogenesis of liver diseases associated with diabetes.

The previous studies in the streptozotocin-induced diabetic rats demonstrated the decreased expression and function of rOAT3 [16, 17], which regulation was likely mediated through impaired insulin signaling involving PKC activities [16, 61]. Consistently, our study also observed the decreased renal expression of mOat3 in the diabetic Ins2Akita mice compared to that of control (Table 3, Fig. 1 and Table 5), which finding confirms that the regulation of Oat3 is insulin dependent. Noteworthy, the altered Oat3 expression and function may potentially impact on anti-diabetic therapies. For example, DA-9801, the herbal preparation currently being evaluated for diabetic peripheral neuropathy in phase II clinical trials, is largely involved with drug-herb interactions. It has been shown to interact with Oat3 and impact on the Oat3-involved cimetidine pharmacokinetic performance [62].

A specific human OCTN1 genetic polymorphism L503F (rs1050152) has been implicated to be correlated with diabetes [63]. However, little evidence has been obtained so far, to show the association between OCTN1 and diabetes. Our study is the first to demonstrate the down-regulated renal expression of mOctn1 in diabetes (Table 3, Fig. 1 and Table 5). Future studies should be warranted to investigate the clinical consequence of such modulation in pathological conditions.

The altered renal expression of rOct1 and rOct2 was reported in the previous study conducted using the streptozotocin-induced diabetic rats [15]. The protein expression of both mOct1 and mOct2 was unchanged in the current study, although the mRNA expression of mOct2 was reduced (Table 3). Such discrepancy might be due to the different characteristics of the invasive streptozotocin-induced rodent model and the Ins2Akita mice model as described above. Interestingly, we found the mRNA and protein expression of mOct3 was increased in the livers of diabetic mice (Table 4, Fig. 2 and Table 6), which transporter has been shown to be closely related to the pharmacokinetic performance and pharmacological effect of the front-line anti-diabetic agent metformin [64, 65]. Therefore, the modulated expression of mOct3 in diabetes should be taken into consideration when administrating metformin and other agents that are specific substrates of this transporter.

Our study also revealed that the renal and hepatic expression of mPept1 and mPept2 was unchanged in diabetic mice compared to that of control, which finding aligns well with the previous report [22].

In summary, taking advantage of the Ins2Akita murine model, our study extensively investigated the mRNA and protein expression of twenty essential SLC transporters in the kidneys and livers of control and diabetic mice. Altered expression of several SLC transporters was observed in diabetic mice compared to that of control. The information gathered in this study could greatly enhance our understanding of the modulation of SLC transporters in pathological conditions, which is likely to impact on the pharmacokinetic performance of drugs that are transported by these transporters and therefore, forms the basis of future therapeutic optimization of regimens in the patients with type 1 diabetes mellitus.

## References

[1] Kaul K, Tarr JM, Ahmad SI, Kohner EM, Chibber R. Introduction to diabetes mellitus. Adv Exp Med Biol. 2012;771:1–11. 23393665 10.1007/978-1-4614-5441-0_1

[2] Janghorbani M, Van Dam RM, Willett WC, Hu FB. Systematic review of type 1 and type 2 diabetes mellitus and risk of fracture. Am J Epidemiol. 2007;166(5):495–505. 17575306 10.1093/aje/kwm106

[3] Giannoukakis N, Pietropaolo M, Trucco M. Therapeutic strategies for Type 1 and Type 2 diabetes mellitus. Diabetes Nutr Metab. 2002;15(3):173–203. 12173733

[4] Patti ME. Gene expression in the pathophysiology of type 2 diabetes mellitus. Curr Diab Rep. 2004;4(3):176–81. 15132881 10.1007/s11892-004-0020-x

[5] Planas R, Pujol-Borrell R, Vives-Pi M. Global gene expression changes in type 1 diabetes: insights into autoimmune response in the target organ and in the periphery. Immunol Lett. 2010;133(2):55–61. 10.1016/j.imlet.2010.08.001 20708640

[6] Dominguez JH, Song B, Maianu L, Garvey WT, Qulali M. Gene expression of epithelial glucose transporters: the role of diabetes mellitus. J Am Soc Nephrol. 1994;5(5 Suppl 1):S29–36. 7873742 10.1681/ASN.V55s29

[7] Roth M, Obaidat A, Hagenbuch B. OATPs, OATs and OCTs: the organic anion and cation transporters of the SLCO and SLC22A gene superfamilies. Br J Pharmacol. 2012;165(5):1260–87. 10.1111/j.1476-5381.2011.01724.x 22013971 PMC3372714

[8] Zhou F, You G. Molecular insights into the structure-function relationship of organic anion transporters OATs. Pharm Res. 2007;24(1):28–36. 17103332 10.1007/s11095-006-9144-9

[9] Smith DE, Clemencon B, Hediger MA. Proton-coupled oligopeptide transporter family SLC15: physiological, pharmacological and pathological implications. Mol Aspects Med. 2013;34(2–3):323–36. 23506874 10.1016/j.mam.2012.11.003 PMC3602806

[10] Nishimura M, Naito S. Tissue-specific mRNA expression profiles of human solute carrier transporter superfamilies. Drug Metab Pharmacokinet. 2008;23(1):22–44. 18305372 10.2133/dmpk.23.22

[11] Nishimura M, Naito S. Tissue-specific mRNA expression profiles of human ATP-binding cassette and solute carrier transporter superfamilies. Drug Metab Pharmacokinet. 2005;20(6):452–77. 16415531 10.2133/dmpk.20.452

[12] DeGorter MK, Xia CQ, Yang JJ, Kim RB. Drug transporters in drug efficacy and toxicity. Annu Rev Pharmacol Toxicol. 2012;52:249–73. 10.1146/annurev-pharmtox-010611-134529 21942630

[13] More VR, Slitt AL. Alteration of hepatic but not renal transporter expression in diet-induced obese mice. Drug Metab Dispos. 2011;39(6):992–9. 10.1124/dmd.110.037507 21430232 PMC3100907

[14] Nowicki MT, Aleksunes LM, Sawant SP, Dnyanmote AV, Mehendale HM, Manautou JE. Renal and hepatic transporter expression in type 2 diabetic rats. Drug Metab Lett. 2008;2(1):11–7. 19356064 10.2174/187231208783478425

[15] Grover B, Buckley D, Buckley AR, Cacini W. Reduced expression of organic cation transporters rOCT1 and rOCT2 in experimental diabetes. J Pharmacol Exp Ther. 2004;308(3):949–56. 14718608 10.1124/jpet.103.058388

[16] Lungkaphin A, Arjinajarn P, Pongchaidecha A, Srimaroeng C, Chatsudthipong L, Chatsudthipong V. Impaired insulin signaling affects renal organic anion transporter 3 (Oat3) function in streptozotocin-induced diabetic rats. PLoS One. 2014;9(5):e96236 10.1371/journal.pone.0096236 24801871 PMC4011703

[17] Phatchawan A, Chutima S, Varanuj C, Anusorn L. Decreased renal organic anion transporter 3 expression in type 1 diabetic rats. Am J Med Sci. 2014;347(3):221–7. 10.1097/MAJ.0b013e3182831740 23470271

[18] Cheng Q, Aleksunes LM, Manautou JE, Cherrington NJ, Scheffer GL, Yamasaki H, et al Drug-metabolizing enzyme and transporter expression in a mouse model of diabetes and obesity. Mol Pharm. 2008;5(1):77–91. 10.1021/mp700114j 18189363

[19] More VR, Wen X, Thomas PE, Aleksunes LM, Slitt AL. Severe diabetes and leptin resistance cause differential hepatic and renal transporter expression in mice. Comp Hepatol. 2012;11(1):1 10.1186/1476-5926-11-1 22524730 PMC3416584

[20] Gangopadhyay A, Thamotharan M, Adibi SA. Regulation of oligopeptide transporter (Pept-1) in experimental diabetes. Am J Physiol Gastrointest Liver Physiol. 2002;283(1):G133–8. 12065300 10.1152/ajpgi.00445.2001

[21] Bikhazi AB, Skoury MM, Zwainy DS, Jurjus AR, Kreydiyyeh SI, Smith DE, et al Effect of diabetes mellitus and insulin on the regulation of the PepT 1 symporter in rat jejunum. Mol Pharm. 2004;1(4):300–8. 15981589 10.1021/mp049972u

[22] Tramonti G, Xie P, Wallner EI, Danesh FR, Kanwar YS. Expression and functional characteristics of tubular transporters: P-glycoprotein, PEPT1, and PEPT2 in renal mass reduction and diabetes. Am J Physiol Renal Physiol. 2006;291(5):F972–80. 17028260 10.1152/ajprenal.00110.2006

[23] Gurley SB, Clare SE, Snow KP, Hu A, Meyer TW, Coffman TM. Impact of genetic background on nephropathy in diabetic mice. Am J Physiol Renal Physiol. 2006;290(1):F214–22. 16118394 10.1152/ajprenal.00204.2005

[24] Qi Z, Fujita H, Jin J, Davis LS, Wang Y, Fogo AB, et al Characterization of susceptibility of inbred mouse strains to diabetic nephropathy. Diabetes. 2005;54(9):2628–37. 16123351 10.2337/diabetes.54.9.2628

[25] Chang JH, Gurley SB. Assessment of diabetic nephropathy in the Akita mouse. Methods Mol Biol. 2012;933:17–29. 10.1007/978-1-62703-068-7_2 22893398

[26] Gurley SB, Mach CL, Stegbauer J, Yang J, Snow KP, Hu A, et al Influence of genetic background on albuminuria and kidney injury in Ins2(+/C96Y) (Akita) mice. Am J Physiol Renal Physiol. 2010;298(3):F788–95. 10.1152/ajprenal.90515.2008 20042456 PMC2838602

[27] Yoshioka M, Kayo T, Ikeda T, Koizumi A. A novel locus, Mody4, distal to D7Mit189 on chromosome 7 determines early-onset NIDDM in nonobese C57BL/6 (Akita) mutant mice. Diabetes. 1997;46(5):887–94. 9133560 10.2337/diab.46.5.887

[28] Wang J, Takeuchi T, Tanaka S, Kubo SK, Kayo T, Lu D, et al A mutation in the insulin 2 gene induces diabetes with severe pancreatic beta-cell dysfunction in the Mody mouse. J Clin Invest. 1999;103(1):27–37. 9884331 10.1172/JCI4431 PMC407861

[29] Wang CP, Wang X, Zhang X, Shi YW, Liu L, Kong LD. Morin improves urate excretion and kidney function through regulation of renal organic ion transporters in hyperuricemic mice. J Pharm Pharm Sci. 2010;13(3):411–27. 21092713 10.18433/j3q30h

[30] Gong L, Aranibar N, Han YH, Zhang Y, Lecureux L, Bhaskaran V, et al Characterization of organic anion-transporting polypeptide (Oatp) 1a1 and 1a4 null mice reveals altered transport function and urinary metabolomic profiles. Toxicol Sci. 2011;122(2):587–97. 10.1093/toxsci/kfr114 21561886

[31] Barthelmes D, Zhu L, Shen W, Gillies MC, Irhimeh MR. Differential gene expression in Lin-/VEGF-R2+ bone marrow-derived endothelial progenitor cells isolated from diabetic mice. Cardiovasc Diabetol. 2014;13:42 10.1186/1475-2840-13-42 24521356 PMC3926942

[32] Surwit RS, Kuhn CM, Cochrane C, McCubbin JA, Feinglos MN. Diet-induced type II diabetes in C57BL/6J mice. Diabetes. 1988;37(9):1163–7. 3044882 10.2337/diab.37.9.1163

[33] Seok SJ, Lee ES, Kim GT, Hyun M, Lee JH, Chen S, et al Blockade of CCL2/CCR2 signalling ameliorates diabetic nephropathy in db/db mice. Nephrol Dial Transplant. 2013;28(7):1700–10. 10.1093/ndt/gfs555 23794669

[34] Kim HJ, Kim BH, Kim YC. Antioxidative action of corni fructus aqueous extract on kidneys of diabetic mice. Toxicol Res. 2011;27(1):37–41. 10.5487/TR.2011.27.1.037 24278549 PMC3834510

[35] Chan T, Zheng J, Zhu L, Grewal T, Murray M, Zhou F. Putative Transmembrane Domain 6 of the Human Organic Anion Transporting Polypeptide 1A2 (OATP1A2) Influences Transporter Substrate Binding, Protein Trafficking, and Quality Control. Mol Pharm. 2014. 10.1021/mp500459b 25387129

[36] Zheng J, Chan T, Cheung FS, Zhu L, Murray M, Zhou F. PDZK1 and NHERF1 regulate the function of human organic anion transporting polypeptide 1A2 (OATP1A2) by modulating its subcellular trafficking and stability. PLoS One. 2014;9(4):e94712 10.1371/journal.pone.0094712 24728453 PMC3984249

[37] Zhou F, Zheng J, Zhu L, Jodal A, Cui PH, Wong M, et al Functional analysis of novel polymorphisms in the human SLCO1A2 gene that encodes the transporter OATP1A2. AAPS J. 2013;15(4):1099–108. 10.1208/s12248-013-9515-1 23918469 PMC3787238

[38] Sakamoto K, Nagamachi Y, Sugawara I. [A new screening method for colorectal cancer as a replacement for the hemoccult blood test]. Gan No Rinsho. 1990;36(8):865–70. 2366322

[39] Toh DS, Murray M, Pern Tan K, Mulay V, Grewal T, Lee EJ, et al Functional analysis of pharmacogenetic variants of human organic cation/carnitine transporter 2 (hOCTN2) identified in Singaporean populations. Biochem Pharmacol. 2011;82(11):1692–9. 10.1016/j.bcp.2011.08.008 21864509

[40] Zhou F, Lee AC, Krafczyk K, Zhu L, Murray M. Protein kinase C regulates the internalization and function of the human organic anion transporting polypeptide 1A2. Br J Pharmacol. 2011;162(6):1380–8. 10.1111/j.1476-5381.2010.01144.x 21133891 PMC3058169

[41] Arabi YM, Dehbi M, Rishu AH, Baturcam E, Kahoul SH, Brits RJ, et al sRAGE in diabetic and non-diabetic critically ill patients: effects of intensive insulin therapy. Crit Care. 2011;15(4):R203 10.1186/cc10420 21871056 PMC3387645

[42] Rett K, Wicklmayr M, Standl E. Hypertension in the non-insulin-dependent diabetes mellitus syndrome: a critical review of therapeutic intervention. J Hypertens Suppl. 1995;13(2):S81–5. 8576794 10.1097/00004872-199508001-00013

[43] Mallat SG. What is a preferred angiotensin II receptor blocker-based combination therapy for blood pressure control in hypertensive patients with diabetic and non-diabetic renal impairment? Cardiovasc Diabetol. 2012;11:32 10.1186/1475-2840-11-32 22490507 PMC3351968

[44] Feghali RE, Nisse-Durgeat S, Asmar R. Effect of candesartan cilexetil on diabetic and non-diabetic hypertensive patients: meta-analysis of five randomized double-blind clinical trials. Vasc Health Risk Manag. 2007;3(1):165–71. 17583187 PMC1994048

[45] Vogt L, Kocks MJ, Laverman GD, Navis G. Renoprotection by blockade of the renin-angiotensin-aldosterone system in diabetic and non-diabetic chronic kidney disease. Specific involvement of intra-renal angiotensin-converting enzyme activity in therapy resistance? Minerva Med. 2004;95(5):395–409. 15467515

[46] Kasichayanula S, Liu X, Pe Benito M, Yao M, Pfister M, LaCreta FP, et al The influence of kidney function on dapagliflozin exposure, metabolism and pharmacodynamics in healthy subjects and in patients with type 2 diabetes mellitus. Br J Clin Pharmacol. 2013;76(3):432–44. 10.1111/bcp.12056 23210765 PMC3769670

[47] Imbert P, Pernod G, Jacquet JP, Bailly C, Laporte S, Lievre M. Evaluation of a mobile electronic assistant to aid in fluindione prescription: the INRPlus cluster randomized trial. Thromb Res. 2014;133(5):756–61. 10.1016/j.thromres.2014.02.003 24582071

[48] Sciullo E, Cardellini G, Baroni MG, Torresi P, Buongiorno A, Pozzilli P, et al Glucose transporter (Glut1, Glut3) mRNA in human placenta of diabetic and non-diabetic pregnancies. Early Pregnancy. 1997;3(3):172–82. 10086067

[49] Zhou F, Zhu L, Cui PH, Church WB, Murray M. Functional characterization of nonsynonymous single nucleotide polymorphisms in the human organic anion transporter 4 (hOAT4). Br J Pharmacol. 2010;159(2):419–27. 10.1111/j.1476-5381.2009.00545.x 20015291 PMC2825363

[50] Kikawa K, Sakano D, Shiraki N, Tsuyama T, Kume K, Endo F, et al Beneficial effect of insulin treatment on islet transplantation outcomes in Akita mice. PLoS One. 2014;9(4):e95451 10.1371/journal.pone.0095451 24743240 PMC3990632

[51] Salem ES, Grobe N, Elased KM. Insulin treatment attenuates renal ADAM17 and ACE2 shedding in diabetic Akita mice. Am J Physiol Renal Physiol. 2014;306(6):F629–39. 10.1152/ajprenal.00516.2013 24452639 PMC3949038

[52] Kidokoro K, Satoh M, Channon KM, Yada T, Sasaki T, Kashihara N. Maintenance of endothelial guanosine triphosphate cyclohydrolase I ameliorates diabetic nephropathy. J Am Soc Nephrol. 2013;24(7):1139–50. 10.1681/ASN.2012080783 23620395 PMC3699824

[53] Durmus S, Naik J, Buil L, Wagenaar E, van Tellingen O, Schinkel AH. In vivo disposition of doxorubicin is affected by mouse Oatp1a/1b and human OATP1A/1B transporters. Int J Cancer. 2014;135(7):1700–10. 10.1002/ijc.28797 24554572

[54] Iusuf D, van Esch A, Hobbs M, Taylor M, Kenworthy KE, van de Steeg E, et al Murine Oatp1a/1b uptake transporters control rosuvastatin systemic exposure without affecting its apparent liver exposure. Mol Pharmacol. 2013;83(5):919–29. 10.1124/mol.112.081927 23429889

[55] Hobbs M, Parker C, Birch H, Kenworthy K. Understanding the interplay of drug transporters involved in the disposition of rosuvastatin in the isolated perfused rat liver using a physiologically-based pharmacokinetic model. Xenobiotica. 2012;42(4):327–38. 10.3109/00498254.2011.625452 22035568

[56] van de Steeg E, van Esch A, Wagenaar E, Kenworthy KE, Schinkel AH. Influence of human OATP1B1, OATP1B3, and OATP1A2 on the pharmacokinetics of methotrexate and paclitaxel in humanized transgenic mice. Clin Cancer Res. 2013;19(4):821–32. 10.1158/1078-0432.CCR-12-2080 23243220

[57] Horiuchi I, Mori YI, Taguchi M, Ichida F, Miyawaki T, Hashimoto Y. Mechanisms responsible for the altered pharmacokinetics of bosentan: analysis utilizing rats with bile duct ligation-induced liver dysfunction. Biopharm Drug Dispos. 2009;30(6):326–33. 10.1002/bdd.671 19639656

[58] Pfefferkorn JA, Guzman-Perez A, Litchfield J, Aiello R, Treadway JL, Pettersen J, et al Discovery of (S)-6-(3-cyclopentyl-2-(4-(trifluoromethyl)-1H-imidazol-1-yl)propanamido)nicotini c acid as a hepatoselective glucokinase activator clinical candidate for treating type 2 diabetes mellitus. J Med Chem. 2012;55(3):1318–33. 10.1021/jm2014887 22196621

[59] Powell DA, Black WC, Bleasby K, Chan CC, Deschenes D, Gagnon M, et al Nicotinic acids: liver-targeted SCD inhibitors with preclinical anti-diabetic efficacy. Bioorg Med Chem Lett. 2011;21(24):7281–6. 10.1016/j.bmcl.2011.10.040 22047692

[60] Zhang Y, Csanaky IL, Cheng X, Lehman-McKeeman LD, Klaassen CD. Organic anion transporting polypeptide 1a1 null mice are sensitive to cholestatic liver injury. Toxicol Sci. 2012;127(2):451–62. 10.1093/toxsci/kfs123 22461449 PMC3355319

[61] Ontawong A, Saowakon N, Vivithanaporn P, Pongchaidecha A, Lailerd N, Amornlerdpison D, et al Antioxidant and renoprotective effects of Spirogyra neglecta (Hassall) Kutzing extract in experimental type 2 diabetic rats. Biomed Res Int. 2013;2013:820786 10.1155/2013/820786 23862157 PMC3686068

[62] Song IS, Kong TY, Jeong HU, Kim EN, Kwon SS, Kang HE, et al Evaluation of the transporter-mediated herb-drug interaction potential of DA-9801, a standardized dioscorea extract for diabetic neuropathy, in human in vitro and rat in vivo. BMC Complement Altern Med. 2014;14:251 10.1186/1472-6882-14-251 25034211 PMC4223725

[63] Nunez C, Santiago JL, Varade J, de la Calle H, Figueredo MA, Fernandez-Gutierrez B, et al IL4 in the 5q31 context: association studies of type 1 diabetes and rheumatoid arthritis in the Spanish population. Immunogenetics. 2008;60(1):19–23. 18064451 10.1007/s00251-007-0265-z

[64] Patel H, Younis RH, Ord RA, Basile JR, Schneider A. Differential expression of organic cation transporter OCT-3 in oral premalignant and malignant lesions: potential implications in the antineoplastic effects of metformin. J Oral Pathol Med. 2013;42(3):250–6. 10.1111/j.1600-0714.2012.01196.x 22861817

[65] Chen L, Pawlikowski B, Schlessinger A, More SS, Stryke D, Johns SJ, et al Role of organic cation transporter 3 (SLC22A3) and its missense variants in the pharmacologic action of metformin. Pharmacogenet Genomics. 2010;20(11):687–99. 10.1097/FPC.0b013e32833fe789 20859243 PMC2976715

